# Microsaccade generation requires a foveal anchor

**DOI:** 10.16910/jemr.12.6.14

**Published:** 2020-05-16

**Authors:** Jorge Otero-Millan, Rachel E. Langston, Francisco Costela, Stephen L. Macknik, Susana Martinez-Conde

**Affiliations:** Johns Hopkins University, Baltimore, MD, USA; University of Arizona, Tucson, AZ, USA; Schepens Eye Research Institute, Boston, MA, USA; State University of New York, Downstate Brooklyn, NY, USA

**Keywords:** microsaccades, scotoma, saccades, natural scenes, free-viewing, artificial scotoma, foveal vision

## Abstract

Visual scene characteristics can affect various aspects of saccade and microsaccade dynamics. For example, blank visual scenes are known to elicit diminished saccade and microsaccade production, compared to natural scenes. Similarly, microsaccades are less frequent in the dark. Yet, the extent to which foveal versus peripheral visual information contribute to microsaccade production remains unclear: because microsaccade directions are biased towards covert attention locations, it follows that peripheral visual stimulation could suffice to produce regular microsaccade dynamics, even without foveal stimulation being present. Here we determined the characteristics of microsaccades as a function of foveal and/or peripheral visual stimulation, while human subjects conducted four types of oculomotor tasks (fixation, free viewing, guided viewing and passive viewing). Foveal information was either available, or made unavailable, by the presentation of simulated scotomas. We found foveal stimulation to be critical for microsaccade production, and peripheral stimulation, by itself, to be insufficient to yield normal microsaccades. In each oculomotor task, microsaccade production decreased when scotomas blocked foveal stimulation. Across comparable foveal stimulation conditions, the type of peripheral stimulation (static versus dynamic) moreover affected microsaccade production, with dynamic backgrounds resulting in lower microsaccadic rates than static backgrounds. These results indicate that a foveal visual anchor is necessary for normal microsaccade generation. Whereas peripheral visual stimulation, on its own, does not suffice for normal microsaccade production, it can nevertheless modulate microsaccadic characteristics. These findings extend our current understanding of the links between visual input and ocular motor control, and may therefore help improve the diagnosis and treatment of ophthalmic conditions that degrade central vision, such as age-related macular degeneration.

## Introduction

Attempts to fasten one’s gaze to a target — either during sustained fixation of a small visual feature, or while scanning large scenes—are known to result in microsaccade production. Microsaccades are jerk-like movements of comparable characteristics to those of larger saccades, and one of three types of fixational eye movements (FEMS), which also comprise intersaccadic drift and tremor (see 1, 2, 3). Whereas the body of research on drift and tremor has been steadily growing, most FEM investigations to date have focused on microsaccades. A main reason has been that microsaccades’ relative larger sizes and speeds allow for their easier measurement and objective characterization via commercial eye trackers and standard detection algorithms (4, 5, 6, 7). 

Thus, research studies have started to address the visual and cognitive influences — which include bottom-up as well as top-down factors — on microsaccade parameters (see 8 for a review). For instance, the size of the fixation target has a substantial impact on microsaccade rates and magnitudes (microsaccade rates decrease linearly and magnitudes increase linearly with target size (9). Yet, as long as the fixation target remains visible, its luminance does not affect microsaccade production (9, 10). 

Scene characteristics also affect microsaccadic dynamics during exploration and search. For example, blank scenes cause decreased (micro) saccadic production during free-viewing, compared with natural scenes (11). Microsaccades are also less frequent in scotopic than in photopic conditions, though their sizes are larger (12, 13). In scenes with visual content, microsaccadic rates are higher on faces vs. non-faces, objects vs. backgrounds, and more informative vs. less informative image regions (11, 14). The size of natural scenes is an additional factor: images of diminishing sizes result in saccade magnitude distributions that shift on a continuum towards smaller saccades, and reach sizes equivalent to those of fixational microsaccades for the smallest images (15). 

Microsaccade production is moreover affected by mental fatigue (16,17) and cognitive load (i.e. task difficulty), even in the absence of visual information (18). Finally, numerous studies have established a strong link between the onset of attention and microsaccade dynamics. Remarkably, microsaccadic rate transiently drops approximately 100–200 ms after the onset of an attentional cue, followed by a transient rate enhancement—a phenomenon known as microsaccadic inhibition (4). 

These influences notwithstanding, there are important gaps that remain in our understanding of how both visual information and viewing task affect microsaccadic features. One such gap relates to the experimental conditions in which observers have access to degraded central visual information and undegraded peripheral visual content. Improved understanding of such scenarios might lead to refinements in the diagnosis and treatment of ophthalmic diseases where central vision is impoverished while peripheral vision is not, such as age-related macular degeneration (19), or even conditions that affect the entire visual field, such as amblyopia (20, 21, 22). In addition, it is not known how the presence of dynamic vs. stationary peripheral information affects microsaccade production. 

Here we set out to answer these questions by measuring microsaccades under four viewing-task conditions, while presenting four types of visual scenes. The viewing conditions consisted on a) a static scene, b) a scene with a simulated scotoma implemented with a gaze contingent display (23, 24, 25, 26), or c) a scene in which either a target or the background moved replicating previously recorded eye movements. 

Previous studies showed links between foveal stimulation and microsaccade production in the context of visual scanning (15) and the correction of eye position errors (27, 28, 29). Our present experimental design allowed us to study (micro)saccade characteristics as a function of foveal and/or peripheral visual stimulation, while assessing the proposal that microsaccades and saccades behave as part of a continuum (11, 15, 30).

## Methods

### Participants

Six subjects (2 females, 4 males) with normal or corrected-to-normal vision participated in the experiments and were paid $15/session. Experiments were carried out under the guidelines of the Barrow Neurological Institute’s Institutional Review Board (protocol number 04BN039) and conformed with the World Medical Association Declaration of Helsinki. Written informed consent was obtained from each subject. 

### Experimental Design 

**Figure 1.**
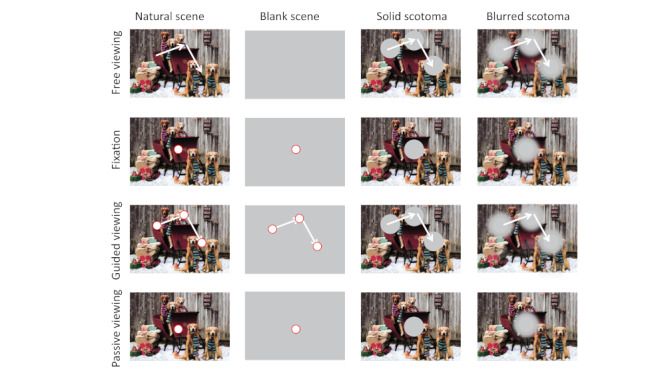
Experimental conditions. The experiments included four viewing conditions (free viewing, fixation, guided viewing, passive viewing) and four scene conditions (natural scene, blank scene, solid scotoma, blurred scotoma). The natural-scene and blank-scene conditions displayed central fixation targets in all viewing conditions, except for the free-viewing condition. In the guided-viewing condition, the fixation target—or the scotoma, when present—moved, replaying the eye movements previously recorded in the free-viewing condition. In the passive-viewing condition, the background moved, replaying the eye movements previously recorded in the free-viewing condition. In the free-viewing scotoma conditions, solid or blurred gaze-contingent scotomas were presented at each fixation location. Fixation targets and scotomas are depicted for illustration purposes, and not to scale.

Subjects rested their foreheads and chins on the EyeLink 1000 head/chin-rest, ~57 cm from a linearized video monitor (Barco Reference Calibrator V monitor, 60 Hz refresh rate, 40x30 cm size, and 1240x1024 pixels resolution). We programmed our experiments in Matlab (The MathWorks, Inc., Natick, MA USA), using the Psychophysics Toolbox extensions (31). Experiments consisted of 5 sessions of ~60 minutes each and tested 4 viewing conditions and 4 scene conditions (Figure 1). Participants completed the experiment over an average of 20 days (4 minimum, 42 maximum), and there was just one subject who performed more than one session per day (two sessions, twice, with a two-hour rest in between sessions).

The viewing (i.e. task) conditions included: 1) a free-viewing condition, 2) a fixation condition, 3) a guided-viewing condition, and 4) a passive-viewing condition. In the free-viewing condition, subjects were instructed to move their eyes freely within the image (eye movements exceeding the area of the image were nevertheless recorded). In the fixation condition, subjects fixated a target formed by two concentric circles, the inner one white (0.25 degrees diameter), and the outer one red (0.5 degrees diameter), placed at the center of the image. The guided-viewing condition was similar to the fixation condition, except that the fixation target jumped around the image, following a pattern determined by the previously recorded free-viewing condition for the same image. In the passive-viewing condition, subjects fixated a static central target, but the image behind it moved, again replaying a pattern determined by the eye movements previously recorded in the free-viewing condition. This replay pattern was not an exact replica of the eye movements recorded beforehand, for two main reasons. First, the previous recordings included not only the eye movements themselves, but also noise (including from blinks and partial blinks, as well as from noise in the image sensor, or from errors in the pupil tracking algorithm, among other possible sources). Second, in the guided-viewing condition subjects had to locate and make a saccade to the target after each target jump, which required additional time to accomplish (as compared to maintaining fixation on a stationary target). Thus, we simplified the replay in both the guided-viewing condition (where the target moved) and in the passive-viewing condition (where the background moved), to consist of a sequence of either target or background jumps, in which consecutive jumps were separated by a minimum period of 900ms, with the target/background completely stationary between jumps.

The scene conditions included: 1) a natural scene condition, 2) a blank scene condition, 3) a solid scotoma condition, and 4) a blurred scotoma condition. The blank scene condition displayed a 50% gray full-screen background. The other three scene conditions displayed natural images from the McGill Calibrated Colour Image Database (32). Subjects saw a total of 15 different natural images, with each image being shown 12 times (4 times in each of the non-blank scene conditions). The image repetitions were intended to reduce experimental error and to enable the comparison of the same set of images across the different viewing tasks. The blank scene condition was similarly presented 4 times. Each image was presented at the center of the screen and covered 768 by 576 pixels, or 24 by 17 degrees of visual angle (note that pixels in our setup where not square). The solid scotoma and the blurred scotoma conditions were as the natural scene condition, except that a simulated scotoma replaced—and served as—the fixation target in each of the viewing tasks. One main goal of this manipulation was to approximate the visual experience of patients suffering from age-related macular degeneration, a condition that results in foveal vision impairment, but which does not affect the rest of the visual field. In the free-viewing task condition, a gaze-contingent scotoma was presented at each fixation location: that is, the position of the scotoma was updated every frame (60 Hz) with the eye position reported by EyeLink through the ethernet link. The delay between an eye movement and the gaze-contingent scotoma movement was estimated between 17 and 34 ms. Because our focus was the analysis of eye movements during fixation periods, where the scotoma remained relatively stable, this delay should not have substantially affected our results.

Both the solid and the blurred scotomas obscured the details of the scene underneath. The solid scotoma was a 50% gray circle with a 3-degree radius. Likewise, the blurred scotoma was a 50% gray circle, but its edges blended smoothly with the background following a gaussian profile. Thus, the scotoma’s transparency was 50% at 4 degrees of distance from its center, and 90% at 6 degrees from its center. Presenting these two types of scotoma enabled us to examine the potential effect of sharp edges on (micro)saccade production (33).

The full experiment included 3 blocks. Each block presented 5 different natural images and consisted of 80 30-s trials that were pseudorandomly interleaved, with the only restriction being that free-viewing trials always preceded replay trials using the same image (the median separation between a replay and its corresponding recording was 42 trials). Thus, each of the 5 experimental sessions contained 48 trials. Each trial was preceded by an instructions screen indicating the task to be performed. 

### Eye Movement Analyses

**Figure 2.**
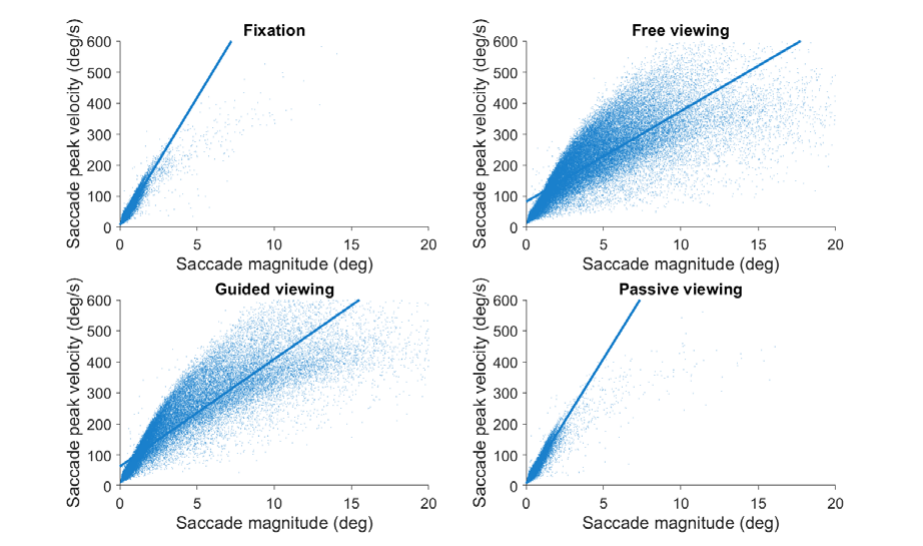
Main sequence relationship between (micro)saccade magnitude and peak velocity in each viewing task.

Eye position was acquired noninvasively in both eyes at 500 samples/s (EyeLink 1000, SR Research). Saccades were identified with a modified version of the algorithm developed by Engbert & Kliegl (4, 30, 34, 35, 36) with λ = 6 (used to obtain the velocity threshold) and a minimum saccadic duration of 6 ms. Microsaccades were defined as saccades with magnitude <1° in both eyes (2, 11, 37). To calculate (micro)saccade properties such as magnitude and peak velocity we averaged the values for the right and left eyes. Figure 2 shows the peak velocity-magnitude relationship, also known as the main sequence (38), for saccades of all sizes, including microsaccades. The analysis included a total of 67,064 saccades. The main sequence serves as a general snapshot of the characteristics of the eye tracking system, allowing for the comparison of analysis methods across studies, and helping to identify potential problems in the data collection or analysis techniques. 

### Statistical Methods

Microsaccade rates were first averaged across images for each condition, and then compared across conditions using a repeated measures ANOVA with two within subject factors: viewing condition and stimulus condition. Statistical significance for post-hoc tests was calculated with t-tests and is indicated in the figures with ** (p<0.01) and *(p<0.05). Summary statistics throughout the article are reported as mean ± standard error of the mean. 

## Results

**Figure 3.**
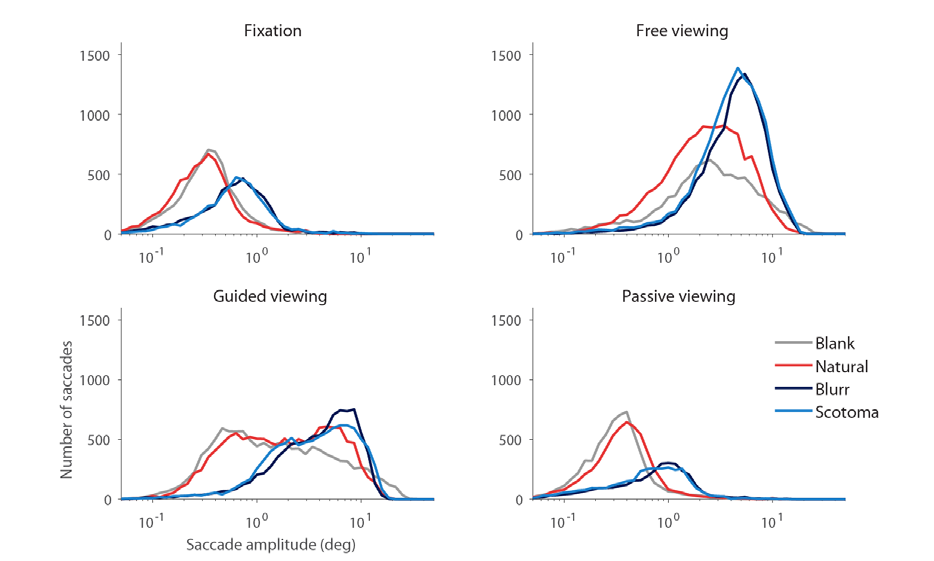
Saccadic amplitude distribution in each experimental condition. Distributions arranged by viewing task condition (N=6 subjects).

**Supplementary figure 1:**
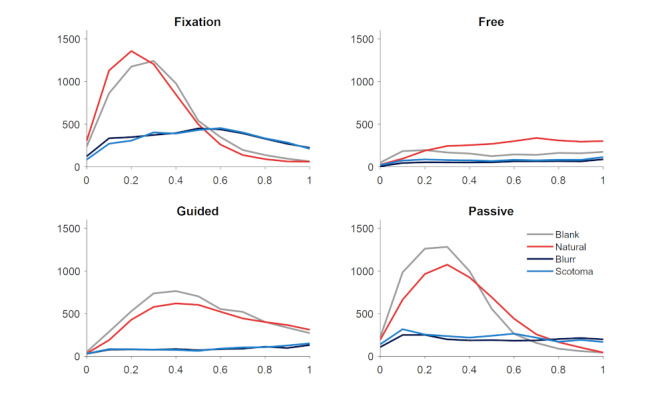
Microsaccadic amplitude distribution in each experimental condition. Distributions arranged by viewing task condition (N=6 subjects).

Subjects observed both blank scenes and natural scenes while conducting four types of oculomotor tasks: fixation, free viewing, guided viewing, and passive viewing. In the guided-viewing condition, subjects fixated a target that replayed the eye movements previously recorded in the free-viewing condition. In the passive-viewing condition, subjects fixated a central target while the background moved, replaying the eye movements previously recorded in the free-viewing condition. Foveal information in natural scenes was either available or made unavailable by the presentation of both solid and blurred scotomas (thus approximating the visual experience of patients with macular degeneration and other foveal impairment conditions). In the free-viewing scotoma conditions, gaze-contingent scotomas were presented at each fixation location (Figure 1). This experimental design allowed us to study the characteristics of microsaccades and saccades as a function of foveal and/or peripheral visual stimulation. Figure 3 shows the amplitude distribution of saccades in each experimental condition, grouped by viewing task and scene condition. Supplementary Figure 1 displays the same data, now focused on the amplitude distribution of microsaccades. Table 1 shows the summary statistics for each condition. Repeated measures ANOVA revealed a significant effect of viewing task (F(1,5)= 38.8, p= 0.002), scene condition (F(1,5)= 45.4, p= 0.001), and their interaction (F(1,5)= 44.5, p= 0.001). Next, we analyze particular comparisons of interest within the dataset.

**Table 1:** Summary statistics of saccade/microsaccade rate and magnitude for all conditions. Values represent mean and standard error of the mean across subjects (N=6).

		Factor 2			
Stim	Task	Microsaccade Rate (1/s)	Microsaccade Magnitude (deg)	Saccade Rate (1/s)	Saccade Magnitude (deg)
Blank Scene	Fixation	1.22 ± 0.24	0.39 ± 0.03	1.3 ± 0.24	0.49 ± 0.05
Blank Scene	Free Viewing	0.34 ± 0.1	0.57 ± 0.03	2.03 ± 0.3	4.14 ± 0.62
Blank Scene	Guided Viewing	0.99 ± 0.12	0.53 ± 0.01	2.42 ± 0.25	3.32 ± 0.48
Blank Viewing	Passive Viewing	1.24 ± 0.24	0.37 ± 0.03	1.31 ± 0.23	0.46 ± 0.04
Blurred Scotoma	Fixation	0.71 ± 0.17	0.59 ± 0.05	0.96 ± 0.14	0.97 ± 0.14
Blurred Scotoma	Free Viewing	0.11 ± 0.04	0.59 ± 0.02	2.68 ± 0.21	5.45 ± 0.43
Blurred Scotoma	Guided Viewing	0.17 ± 0.05	0.56 ± 0.05	1.91 ± 0.15	5.36 ± 0.39
Blurred Scotoma	Passive Viewing	0.4 ± 0.1	0.56 ± 0.04	0.67 ± 0.09	1.21 ± 0.13
Natural Scene	Fixation	1.23 ± 0.27	0.37 ± 0.03	1.31 ± 0.27	0.47 ± 0.06
Natural Scene	Free Viewing	0.51 ± 0.04	0.61 ± 0.01	2.89 ± 0.19	3.34 ± 0.15
Natural Scene	Guided Viewing	0.87 ± 0.09	0.56 ± 0.03	2.6 ± 0.12	3.27 ± 0.14
Natural Scene	Passive Viewing	1.11 ± 0.14	0.42 ± 0.04	1.18 ± 0.14	0.54 ± 0.07
Solid Scotoma	Fixation	0.73 ± 0.17	0.57 ± 0.03	0.96 ± 0.17	0.94 ± 0.13
Solid Scotoma	Free Viewing	0.15 ± 0.04	0.51 ± 0.02	2.93 ± 0.23	5.41 ± 0.36
Solid Scotoma	Guided Viewing	0.18 ± 0.04	0.59 ± 0.03	1.89 ± 0.13	5.24 ± 0.27
Solid Scotoma	Passive Viewing	0.46 ± 0.13	0.54 ± 0.04	0.7 ± 0.11	1.17 ± 0.2

### Effects of present versus absent foveal stimulation on microsaccades

Natural scenes with (solid and blurred) scotomas occluding the center of vision provided intact peripheral stimulation without foveal information. In contrast, blank scene conditions provided deprived peripheral stimulation, either in the presence of a foveal fixation target (in the fixation, guided-viewing, and passive-viewing tasks) or in its absence (in the free-viewing task). Finally, natural scenes without scotomas provided intact information throughout the visual field. 

**Figure 4.**
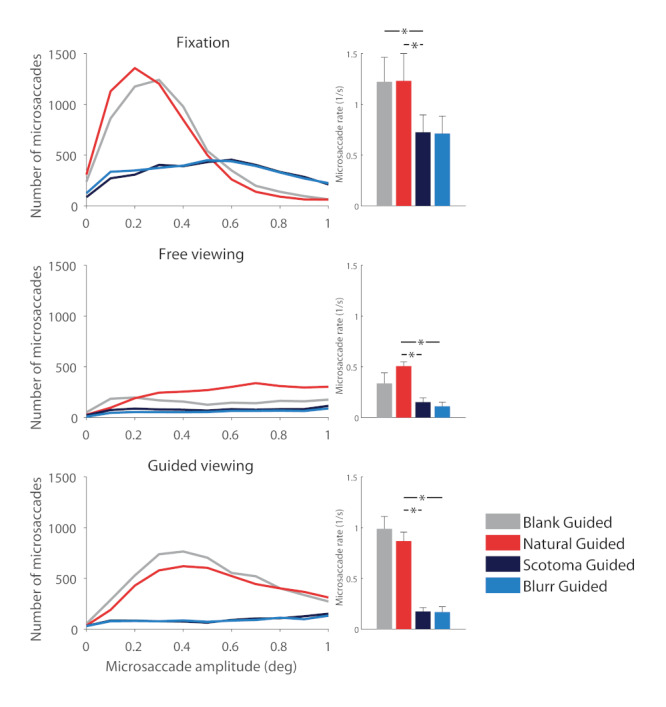
Effect of present vs. absent foveal stimulation on microsaccade production during fixation (top row), free viewing (middle row) and guided viewing (bottom row). Left column: Microsaccade amplitude distributions for each experimental condition, for all subjects combined. Right column: Average microsaccade rate across subjects. Overall, the solid scotoma and blurred scotoma conditions resulted in lower microsaccade rates than the natural scene and blank scene conditions. Error bars indicate the standard error of the mean (N=6 subjects). Significant comparisons are highlighted with an asterisk (p<0.05).

 Our data revealed that, in the absence of a foveal stimulus to fixate on, observers produced fewer microsaccades. Figure 3 shows that the presence of either type of scotoma, by nulling foveal stimulation, had the effect of shifting saccadic magnitudes towards larger values, during fixation, free-viewing, and guide-viewing tasks. Figure 4 takes a closer look at microsaccades produced under the same viewing conditions, and illustrates their dramatic decrease in number in the presence of foveal scotomas.

When subjects fixated a central target presented over a blank or a natural scene, their microsaccade rates were 1.2±0.2 and 1.2±0.3 microsaccades/s respectively. When these fixation targets were replaced by either solid or blurred scotomas, microsaccade rates dropped to 0.7±0.2 microsaccades/s (t(5) =3.2 , p = 0.02). The natural and blank scene free-viewing conditions produced 0.50±0.04 and 0.3±0.1 microsaccades/s respectively, in agreement with prior reports of lower microsaccadic rates during free exploration of blank vs. natural scenes (11). The introduction of solid and blurred scotomas on natural scenes further decreased microsaccade production during visual exploration, to 0.15±0.04 and 0.11±0.04 microsaccades/s respectively (from 0.5±0.04 microsaccades/s; t(5)=14.3, p<0.0001). During guided-viewing conditions, where the fixation target (or the scotoma, when present) moved to replay previously recorded eye movements, natural scenes produced 0.86±0.08 microsaccades/s, blank scenes 0.98±0.1 microsaccades/s, solid scotomas 0.17±0.04 microsaccades/s, and blurred scotomas 0.17±0.05 microsaccades/s (t=6.1,p =0.0008). 

### Effects of dynamic versus static peripheral stimulation on microsaccades

The fixation condition allowed us to study microsaccade characteristics in the presence of static peripheral stimulation. The passive-viewing condition—in which observers fixated either a central fixation target or a central scotoma while the background replayed previously recorded eye motions—allowed us to study microsaccades characteristics in the presence of dynamic peripheral stimulation. The guided viewing task, when conducted over natural scenes, moreover allowed us to study microsaccades in the presence of stationary peripheral stimulation that was nevertheless continuously updated by gaze shifts. The same guided viewing task, when conduced over a blank scene, provided stationary albeit deprived peripheral stimulation. 

**Figure 5.**
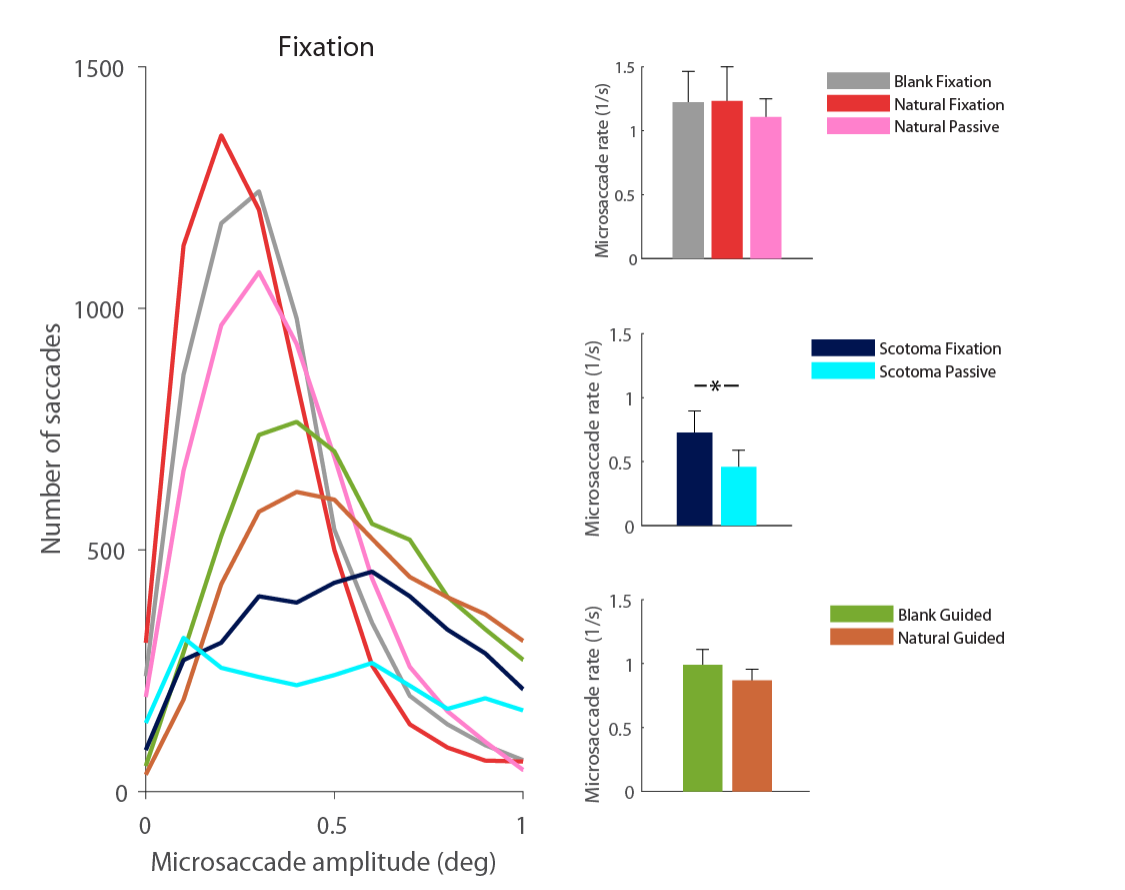
Effect of peripheral stimulation on microsaccades. Left panel: microsaccade amplitude distributions for each experimental condition, for all subjects combined (N=6). Right column: average saccade rate across subjects, for a selection of comparisons. Dynamic peripheral stimulation tended to suppress microsaccades when compared to static peripheral stimulation. This was true for the fixation of a static natural scene vs. a dynamic natural scene (i.e. during passive viewing, top) for the fixation of a scotoma with a static background vs. a dynamic background (middle), or for the guided viewing of a fixation target over a blank scene vs. the guided viewing of a fixation target over a static natural scene (in which the background did not move, but large eye movements repeatedly “refreshed” the peripheral retinal input) (bottom). Significant comparisons are highlighted with an asterisk (p<0.05).

Our data revealed a decrease in microsaccade production in the presence of dynamic peripheral stimulation (i.e. during passive viewing), compared to that recorded with static peripheral stimulation (i.e. during non-passive viewing scenarios). Pairwise comparisons showed that dynamic peripheral stimulation tended to suppress microsaccade generation in a variety of conditions, including: a) central fixation of a static natural scene vs. a dynamic natural scene (i.e. passive viewing) (t=1.6, p=0.07), b) fixation of a scotoma with a static background vs. a dynamic background (i.e. passive viewing) (t=2.0, p=0.05), and c) guided viewing of a fixation target over a blank scene vs. guided viewing of a fixation target over a natural scene (in which the background did not move, but large eye movements repeatedly updated the peripheral retinal input) (t=0.9, p=0.2) (Figure 5).

**Figure 6.**
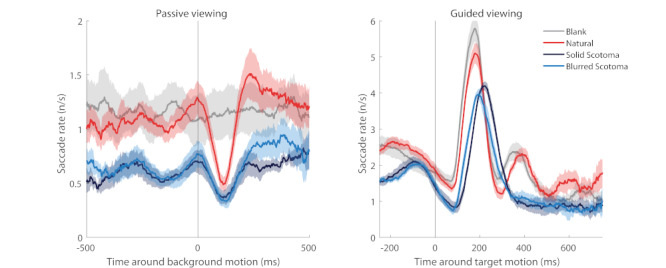
Correlation between image motion and (micro)saccade production in the passive-viewing and guided-viewing conditions. Each line indicates the average (micro)saccade rate across subjects relative to the occurrence of peripheral image motion (left panel) or fixation target motion (right panel). The lines where smoothed with a Savitzky-Golay filter of length 100 ms. Shaded areas represent the standard error of the mean across subjects (N=6).

We wondered if some of the differences above could be related to the known transient suppression of microsaccade production that takes place shortly after peripheral stimulation changes (4, 34). To find out, we correlated the onsets of either motions of the background image (corresponding to saccades in passive viewing) or those of the fixation target (corresponding to saccades in guided viewing) to the onsets of microsaccades. Figure 6 shows the results for each type of condition. In passive viewing (Figure 6 left), all conditions displaying an actual stimulus resulted in the suppression of (micro)saccades at ~120 ms after the background image motion. This inhibition was more pronounced in the case of natural images than in the scotoma conditions, given that microsaccade production was already depressed in the latter ones, due to the absence of foveal stimulation. In the case of the blank scene, there was no actual motion of the uniform image background; therefore, there was no resulting (micro)saccadic inhibition. 

In guided viewing (Figure 6 right), the fixation target motion led to a large increase in saccade production in all scene conditions, followed by a smaller saccadic bump in the foveal stimulation conditions only. Because this secondary boost presumably reflects the occurrence of microsaccades and corrective saccades in the presence of foveal inputs, it did not arise in either scotoma condition.

## Discussion

Here we set to determine the differential effect of foveal vs. peripheral visual stimulation on microsaccade production. We tested different viewing tasks (fixation, free viewing, guided viewing, passive viewing) in combination with various visual stimuli (blank scenes, static natural scenes, dynamic natural scenes), and the presence/absence of two different types of simulated foveal scotomas. We found that microsaccade production was reduced in the absence of foveal stimulation in every type of viewing task, supporting the proposal that microsaccades require the presence of a “target to anchor to” (11). The effect of peripheral stimulation on microsaccade production was overall suppressive, though only significant in cases where the background’s motion/updating served to renew the peripheral visual input. 

### Effects of simulated visual loss on exploratory eye movements

Previous studies have explored the effect of simulated scotomas on eye movements during visual exploration (26, 39, 40, 41). Laubrock and colleagues characterized eye movements during visual exploration of natural scenes, using gaze contingent displays to apply different filters to the foveal or peripheral stimulation. Though their study focused on the effects of the different kinds of stimulation on fixation durations, they also found that low pass filtering of foveal stimulation reduced the production of saccades smaller than 1 degree during visual exploration. In contrast, low pass filtering of the visual periphery did not alter the distribution of saccades below 1 degree. David et al. (2018) tested the effects of peripheral and foveal masking on saccade amplitudes, and found a shift towards larger saccade sizes when foveal masks were applied. The larger the foveal mask, the greater the shift towards larger saccades, possibly because the bigger masks forced subjects to find new objects of interest at greater retinal eccentricities. McIlreavy et al. (2012) found no major changes in saccadic amplitudes in the presence of an artificial scotoma, but their data suggests a reduction in the occurrence of smaller saccades. This is consistent with our observation of a decreased microsaccade production during visual exploration in the absence of foveal input.

### Effects of varying foveal visual stimulation on microsaccades

Prior research has investigated how the characteristics of fixation targets affect microsaccadic features during sustained fixation (42, 43). These studies found that, as long there was a fixation target, microsaccades occurred during fixation attempts. In addition, most target features had minor or negligible effects on microsaccade dynamics, except for target size, which did substantially affect both microsaccade rates and sizes. Thus, Thaler et al. varied the shape of fixation targets and found that microsaccades rates were comparable across conditions, ranging from 1.6 to 1.8 microsaccades/s. Mccamy et al. reported that, whereas the absence of a fixation target reduced the frequency of microsaccades and increased their amplitude, the contrast of visible targets had little effect on microsaccade dynamics. This same study found that the size of the fixation target (a solid circular fixation spot varying in diameter from ~0.07 to 2 deg) had a substantial impact on microsaccade production, however, with bigger targets resulting in larger and less frequent microsaccades (42, 43).

In the current study, participants were instructed to fixate the centers of foveal scotomas whenever present, raising the possibility that such scotomas may have acted as enlarged fixation targets for all practical purposes. If so, the reduced microsaccade rates and distribution shifts towards larger microsaccade magnitudes, observed in our two scotoma conditions, may not only be compatible with, but also extend, the earlier results by McCamy et al.—albeit with even larger fixation targets. Another way to interpret McCamy et al.’s findings in light of the present data is that large fixation targets work like simulated scotomas, in that they deprive vision of fine-grained information at the very center of gaze.

### Effects of ophthalmic loss on microsaccades

Patients affected with macular disease can suffer from localized vision loss, resulting in foveal scotomas. Previous studies (39, 41) found that the distributions of microsaccade sizes in patients with macular disease shifted towards larger magnitudes, consistent with our present results with simulated scotomas. Contrary to our current findings, however, microsaccade rates in macular degeneration patients were comparable to those in healthy participants (19). One important difference between vision with a pathological versus a simulated scotoma is that patients with macular disease often develop a non-foveal preferred retinal locus (PRL). That is, they tend to fixate their gaze using a part of the retina other than the fovea. In contrast, our subjects would not be expected to develop a PRL, given that trials with and without foveal stimulation were randomly interleaved, and that foveal stimulation was only lacking for an average of 13.5 min per session (instead of the hours previously reported to be necessary to develop a PRL (44)). Ophthalmic conditions in which the visual loss extends to the whole visual field (rather than just the fovea), such as amblyopia, may not result in a PRL either, but they do nevertheless affect microsaccade production. Thus, patients with amblyopia display increased overall fixation instability (20, 22), which is accompanied by larger but less frequent microsaccades (22, 45). We note that this pattern of microsaccade production in amblyopia resembles that obtained in our current study when healthy participants fixated either the center of a blank scene or a scotoma. However, it could not be induced in previous research by merely blurring the visual input (46), perhaps because amblyopia’s sustained visual loss has a greater impact on fixational dynamics than transiently blurring a visual scene. 

### Link between peripheral visual stimulation and microsaccade production

We found more frequent microsaccades when participants fixated a target that was presented over a static background than over a dynamic background. This difference was further enhanced when a simulated scotoma blocked all foveal stimulation and subjects were instructed to fixate at the center of the empty region. Our findings are consistent with, and extend in new directions, prior work showing that the sudden onsets of peripheral visual stimuli transiently suppress microsaccade production (4, 34). The time course of this phenomenon, known as microsaccadic inhibition, or the ‘microsaccade signature,’ consists of a phasic response with a microsaccade rate reduction around 100 to 200 ms after the peripheral stimulus onset, sometimes followed by a secondary increase shortly afterwards (47, 48). Our present findings might be considered a variation of this effect, in which each jump of the image acts as a new peripheral stimulation onset, leading to the observed overall microsaccade rate reduction.

### Conclusions

Microsaccades are generated during fixation in response to foveal stimulation, and used by the visual system to scan visual targets features (15) and correct eye position errors (27, 28, 29). Whereas peripheral stimulation can modulate microsaccade dynamics, it does not seem essential for microsaccade generation. Our combined results support the proposal that microsaccade production requires the presence of an anchoring target (11). These findings deepen our understanding of the links between visual inputs and ocular motor control, and may aid the diagnosis and treatment of ophthalmic conditions that result in foveal impairment, such as age-related macular degeneration. Future studies may be conducted with larger populations and image sets, and thus avoid any potential biases inadvertently introduced in our design.

## Ethics and Conflict of Interest

The author(s) declare(s) that the contents of the article are in agreement with the ethics described in http://biblio.unibe.ch/portale/elibrary/BOP/jemr/ethics.html and that there is no conflict of interest regarding the publication of this paper.

## Acknowledgments 

We thank Andrew Danielson, Behrooz Kousari, and Peter Wettenstein for technical assistance, and Hector Rieiro, Dr. Xoana G. Troncoso, and Dr. Michael B. McCamy for their comments. This work was supported by the National Eye Institute (Award K99EY027846 to JOM) and the National Science Foundation (Awards 1523614 to SLM and 1734887 to SLM and SMC).
